# High Frequency Occult Hepatitis B Virus Infection Detected in Non-Resolved Donations Suggests the Requirement of Anti-HBc Test in Blood Donors in Southern China

**DOI:** 10.3389/fimmu.2021.699217

**Published:** 2021-07-28

**Authors:** Xianlin Ye, Yu Zhao, Ran Li, Tong Li, Xin Zheng, Wen Xiong, Jinfeng Zeng, Min Xu, Limin Chen

**Affiliations:** ^1^Department of Laboratory, Shenzhen Blood Center, Shenzhen, China; ^2^Provincial Key Laboratory for Transfusion-Transmitted Infectious Diseases, Institute of Blood Transfusion, Chinese Academy of Medical Sciences (CAMS) and Peking Union Medical College (PUMC), Chengdu, China; ^3^The Joint Laboratory on Transfusion-Transmitted Diseases (TTD) Between Institute of Blood Transfusion, Nanning Blood Center, Chinese Academy of Medical Sciences and Nanning Blood Center, Nanning, China; ^4^Toronto General Research Institute, University Health Network, University of Toronto, Toronto, ON, Canada

**Keywords:** blood safety, occult hepatitis B infection, nucleic acid testing, anti-HBc, minipool

## Abstract

**Background:**

Most Chinese Blood Centers adopted mini pool (MP) nucleic acid testing (NAT) for HBV screening due to high cost of Individual donation (ID) NAT, and different proportions of MP-reactive but ID-non-reactive donations (MP+/ID−, defined as non-resolved donations) have been observed during daily donor screening process. Some of these non-resolved donations are occult HBV infections (OBIs), which pose potential risk of HBV transmission if they are not deferred. This study is aimed to further analyze these non-resolved donations.

**Methods:**

The non-resolved plasma samples were further analyzed by serological tests and various HBV DNA amplification assays including quantitative PCR (qPCR) and nested PCR amplifying the basic core and pre-core promoter regions (BCP/PC; 295 base pairs) and HBsAg (S) region (496 base pairs). Molecular characterizations of HBV DNA+ non-resolved samples were determined by sequencing analysis.

**Results:**

Of 17,226 MPs from 103,356 seronegative blood donations, 98 MPs were detected reactive for HBV. Fifty-six out of these 98 (57.1%) reactive MPs were resolved as HBV DNA+, but the remaining 42 pools (42.9%, 252 donations) were left non-resolved with a high rate (53.2%) of anti-HBc+. Surprisingly, among 42 non-resolved MPs, 17 contained one donation identified as OBIs by alternative NAT assays. Sequence analysis on HBV DNAs extracted from these OBI donations showed some key mutations in the S region that may lead to failure in HBsAg detection and vaccine escape.

**Conclusion:**

A total of 53.2% of the non-resolved donations were anti-HBc+, and OBIs were identified in 40.5% of these non-resolved pools. Therefore, non-resolved donations with anti-HBc+ might pose potential risk for HBV transmission. Our present analysis indicates that anti-HBc testing in non-resolved donations should be used to identify OBIs in order to further increase blood safety in China.

## Introduction

Occult hepatitis B virus (HBV) infection (OBI) is characterized by the presence of very low levels of HBV DNA in the plasma and/or in the liver, with undetectable hepatitis B surface antigen (HBsAg), with or without antibodies to hepatitis B core antigen (anti-HBc) or hepatitis B surface antibody (anti-HBs), outside the pre-seroconversion window period (1), and OBIs may contribute to the exacerbation of acute HBV infection and the development of HBV-associated cirrhosis and hepatocellular carcinoma (HCC) (2). OBI has been found in blood donors, and the prevalence varies from less than 1 to 16% dependent on the endemicity of HBV infection, the screening NAT assays, and confirmation algorithms used (3–6). Although donations with OBI pose a significant risk of transfusion-transmitted HBV infection, OBI screening is very difficult due to intermittent very low virus load and mutations in HBV genome (7, 8).

The blood screening strategy by employing both HBsAg and anti-HBc detection combined with HBV NAT assay in some developed countries allows the detection of window-period infection, OBI, and HBV mutated strains possible to maximally ensure blood safety. However, in HBV endemic countries such as in China, deferral of all anti-HBc positive donations was impractical due to the shortage of blood supply. In addition, high cost of ID-NAT prompt users to adopt screening strategies of pooling of multiple donor samples (9). As a pilot NAT project initiated by the National Health Commission (NHC) of China, Shenzhen Blood Center has been using the Roche Cobas TaqScreen MPX test2.0 for HBV DNA, hepatitis C virus (HCV) RNA, and human immune-deficiency virus (HIV) RNA in routine screening in MP6 (pooled from six individual donations) format since 2017. It turns out this assay is highly specific, sensitive, reproducible, and robust (10, 11). However, different proportions of MP-reactive but individual non-reactive donations (MP+/ID−) have been observed during daily screening. In ID-NAT screening, non-repeat-reactive (NRR) donations are not released for transfusion in most countries. However, for MP6-NAT it is not an option to discard the units implicated in non-resolved pools, leaving a fatal threat to blood safety, particularly in HBV high prevalent countries such as China.

Previously, we have reported nearly half of the initial reactive, but further discriminatory test negative donations were identified as OBIs, which strongly suggested that a proportion of donations with ID-NAT screening NRR results might pose HBV transmission risk even when using highly sensitive NAT assays (12). In this study, we move on to further analyze the non-resolved reactive mini pools in respect to their potential transmission risk for HBV infection. In order to clarify and evaluate the true infection status of the non-resolved donations, alternative Ultrio Plus ID-NAT, nested PCR for S, BCP/PC, and qPCR with high volume extraction were performed (13), and HBV infection status will be characterized from these non-resolved donations. The optimized screening strategy by adding anti-HBc testing will also be discussed.

## Materials and Methods

### Subjects and Samples

As a routine practice, all donations were collected and screened serologically as reported in our previous study (12). In total, 103,356 seronegative blood samples (17,226 MP6 pools) were enrolled in this study for further analysis.

### NAT Screening of Donor Samples

Six individual donated blood were pooled into one mini pool (MP6), and NAT screening of the MP6 was performed on a fully automated Roche Cobas s 201 system using a multiplex polymerase chain reaction kit (Cobas TaqScreen MPX test, version 2.0, Roche Molecular Systems, Branchburg, NJ, USA; 1 ml, LOD: 2.3 IU/ml for HBV DNA, 1 IU/ml=5.6 copies/ml). Donations with negative NAT results were released, and MPX-reactive MP6 were resolved by retesting each individual donation (ID-NAT). Samples individually reactive were classified as MPX repeat reactive (designated as NAT+). If one NAT+ was identified, other five non-reactive donations were designated as NAT− and can be released. If all six individual donations in the pool were detected as MPX non-reactive, they were classified as MP non-resolved donations. These non-resolved donations were further tested individually by an alternative Procleix Ultrio Plus HBV discriminatory assay (Grifols Diagnostic Solutions, Inc. and Hologic; 0.5 ml, LOD: 3.4 IU/ml) to identify the presence of HBV DNA. Donations showing non-reactivity in the Ultrio Plus assay were classified as NAT non-resolved donations by Ultrio Plus MPX. Donations showing reactivity in discriminatory HBV assays were identified as HBV NAT+, and the pool was regarded as resolved pool by Ultrio Plus.

### Serological and Molecular Detection

All resolved and non-resolved samples, including frozen plasma, were collected for further determination. HBsAg, anti-HBs, HBeAg, anti-HBe, and anti-HBc were tested by commercially available electrochemiluminescence immunoassay, ECLI (Roche, USA). All samples were retested on anti-HBc by a domestic EIA kits (WanTai Diagnostics, Beijing, China), and only samples with reactivity for both assays were designated as anti-HBc+.

HBV DNA was additionally extracted from 2.5 ml of plasma by HighPure Viral Nucleic Acid Large Volume Kits (Roche Diagnostics Gmbh, Mannhein, Germany) and were analyzed by a combination of qPCR (13) (LOD: 5 IU/ml) and nested PCRs (the LOD for the combination qPCR/nested PCR assay used with high volume extraction can reach as low as 1 IU/ml (5.6 copies/ml)), which amplified BCP/PC (LOD: 10 IU/ml) and S regions (LOD: 10 IU/ml) as previously described (4, 14).

### HBV DNA Sequencing and Genotyping

The amplified products of BCP/PC and S regions were sent to Shanghai Invitrogen Co., Ltd. (Guangzhou, China) for sequencing. HBV genotyping was performed by Phylogenetic analysis using MEGA7.1 program, and the neighbor-joining method based on Kimura 2-parameter mode and complete deletion for gaps with 1,000 bootstrap replications was chosen as previously reported (13). So-called wild type consensus sequences were derived from the alignment of 124 genotype B and 95 genotype C sequences from HBsAg+ blood donors (4).

### Statistical Analyses

Stata 16.0 was applied for statistical analysis of various data. *P* < 0.05 (two-tailed) was set as the significant cut-off level.

## Results

### Screening Results of the Blood Donations

**Figure 1:**
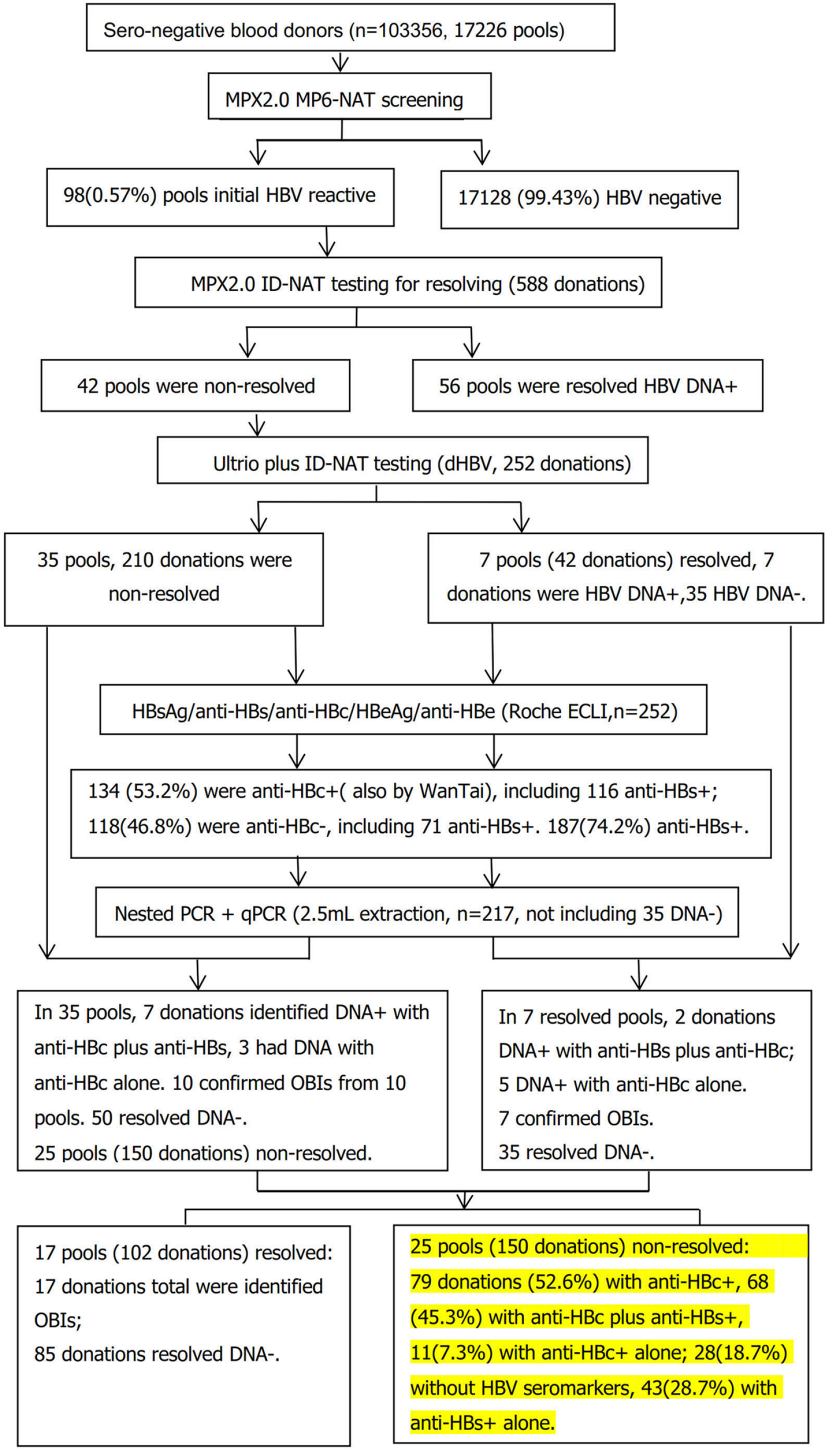
Flow chart of serological and molecular identification of MPX 2.0 MP6-NAT in non-resolved samples.

A total of 103,356 seronegative blood donations were collected. A total of 17,226 pools were derived, and 98 pools were reactive for HBV DNA by MPX2.0 NAT in MP6 format. Fifty-six pools (56/98, 57.1%) were resolved with HBV DNA+ (MP+/ID+), and 42 pools (42.9%, 252 donations) were detected non-reactive by MPX2.0 ID-NAT (MP+/ID−, **Figure 1**), including 169 males and 83 females (**Table 1**). The first-time and repeat donors were 115 (45.6%) and 137 (54.4%), respectively.

**Table 1:** Demographic and viral characteristics of 252 (42 pools) non-resolved blood donations.

	Total (%)	Anti-HBc+ (%)	Anti-HBs (IU/L)			HBV DNA+ (%)
			Negative (%)	10–100	>100	
	252 (100)	134 (53.2)	65 (25.8)	80 (31.7)	107 (42.5)	17 (6.7)
Gender						
Male	169 (67)	92 (54.4)	43 (25.4)	55 (32.5)	71 (42.0)	9 (5.3)
Female	83 (33)	42 (50.6)	22 (26.5)	25 (30.1)	36 (43.4)	8 (9.6)
P-value		NS	NS	NS	NS	NS
Donor type						
First time	115 (45.6)	58 (50.4)	25 (21.7)	42 (36.5)	48 (41.7)	7 (6.1)
Repeat donors	137 (54.4)	76 (55.5)	40 (29.2)	38 (27.7)	59 (43.1)	10 (7.3)
P-value		NS	NS	NS	NS	NS
Age groups						
18–30	96 (38.1)	31 (32.3)	24 (25.0)	31 (32.3)	41 (42.7)	2 (2.1)
31–40	76 (30.2)	42 (55.3)	24 (31.6)	21 (27.6)	31 (40.8)	6 (7.9)
41–50	56 (22.2)	42 (75.0)	11 (19.6)	23 (41.1)	22 (39.3)	7 (12.5)
51–60	24 (9.5)	19 (79.2)	6 (25.0)	5 (20.8)	13 (54.2)	2 (8.3)
P-value		0.00	NS	NS	NS	NS*

*The rate of HBV DNA+ in the 18–30 age group is significantly lower than that in the 30–60 age group (P < 0.05); anti-HBs negative: <10 (IU/L). MPX2.0 (HBV ID-NAT), Ultrio Plus dHBV, BCP/PC (nested PCR) and S (nested PCR): diagnose HBV DNA positivity. Virus loads determined by qPCR. NS, nonsignificance.

### Supplemental Serological Testing Results for 252 Non-resolved Donations

After tested by Elecsys II assay for HBsAg, anti-HBs, HBeAg, anti-HBe, and anti-HBc, 134 out of the 252 non-resolved donations (53.2%) were reactive for anti-HBc, all of which were confirmed positive by WanTai anti-HBc kit. There was no correlation between the presence of anti-HBc and gender or donor type. In contrast, there was a clear increase of anti-HBc prevalence with age, ranging from 32.3% in the <30 age group to 79.2% in the >50 age group (*χ2 =* 34.2, p=0.00).

### Ultrio Plus ID-NAT, Nested PCR, and qPCR Testing for HBV DNA of Non-Resolved Donations

HBV DNA was further analyzed individually for the 252 non-resolved donations by Ultrio plus dHBV, and seven donations out of 42 MPs (252 donations) (7/252 = 2.8%) were resolved as dHBV+, leaving 210 donations still non-resolved. After the 210 non-resolved samples and 7 dHBV+ samples were further analyzed by nested PCR (BCP/PC and S region) and qPCR with 2.5 ml extraction of DNA individually, 10 donations out of 35 MPs (150 donations) were identified as HBV DNA+ (**Table 2**). All together, 17 donations from 42 MPs (252 donations) were resolved HBV DNA+ (one per pool).

**Table 2:** The serological and molecular characterization results of the 17 identified HBV DNA+ donations.

Samples	Gender	Age	Times	HBsAg (IU/ml)	Anti-HBs (IU/L)	Anti-HBc	HBeAg	Anti-HBe	MPX2.0	Ultrio Plus dHBV	BCP/PC	S	Virus load (IU/ml)	Genotype
Q8	F	30	1	<0.05	<2.00	+	–	+	–	–	+	+	–	B
Q15	M	47	8	<0.05	22.31	+	–	–	–	–	–	+	–	C
Q27	F	52	2	<0.05	3.88	+	–	–	–	–	+	+	–	B
Q42	F	25	1	<0.05	2.42	+	–	–	–	–	–	–	6.7	/
Q52	F	38	1	<0.05	>1,000	+	–	–	–	–	–	–	9.1	/
Q61	M	35	1	<0.05	233.6	+	–	–	–	–	–	–	6.6	/
Q84	F	50	22	<0.05	7.43	+	–	–	–	–	–	+	–	B
Q95	M	37	1	<0.05	99.22	+	–	–	–	–	+	–	–	/
Q120	M	34	2	<0.05	100.6	+	–	–	–	–	–	–	7.2	/
Q139	F	47	17	<0.05	39.23	+	–	–	–	–	–	+	–	B
L007	M	51	18	<0.05	531	+	–	–	–	+	–	+	–	D
L012	M	33	1	<0.05	4.9	+	–	–	–	+	–	+	12.1	B
L017	M	32	1	<0.05	<2.0	+	–	–	–	+	–	+	6.5	B
L019	M	43	1	<0.05	<2.0	+	–	–	–	+	–	–	5.1	/
N005	F	45	2	<0.05	30	+	–	–	–	+	+	–	36.2	/
N022	F	47	1	<0.05	<2	+	–	–	–	+	+	+	–	B
N023	M	41	1	<0.05	3.15	+	–	–	–	+	+	–	37.2	/

BCP/PC, basic core promoter/pre-core; F/R, first-time donors/repeat donors; HBV, hepatitis B virus; anti-HBe, antibody to hepatitis B virus e antigen; HBeAg, hepatitis B e antigen; ID, individual donation; dHBV, Procleix Ultrio plus HBV discriminatory assay. MPX2.0 (HBV ID-NAT), Ultrio Plus dHBV, BCP/PC (nested PCR) and S (nested PCR): diagnose HBV DNA positivity. Virus loads determined by qPCR.

Of the 17 identified HBV DNA positive donors, seven were repeat donors and 10 were first-time donors, and eight were males and nine were females with average age of 36.2. Regarding the other viral markers, eight had anti-HBc alone, one carried anti-HBc and anti-HBe, and eight were positive for both anti-HBc and anti-HBs. Three out of the eight anti-HBs positive samples had titers over 100 IU/L. Finally, 150 donations still remained non-resolved, among which 17 were identified as OBIs, and 85 donations were resolved HBV DNA negative.

### Comparison of Seromarkers Distribution Among Non-Resolved 252 Donations, Remaining 150 Non-Resolved Donations, and Resolved Negative Donations

In the remaining non-resolved donations, 79/150 (52.6%) donations carried anti-HBc, in which 11 cases (7.3%) were anti-HBc alone. While, in the resolved negative donations from 17 resolved pools (85 donations), 38/85 (44.7%) donations carried anti-HBc, including 1 (1.2%) with anti-HBc alone (**Table 3**). The rate of anti-HBc alone in the remaining non-resolved donations is six times higher than that in the resolved negative donations (P<0.05).

**Table 3:** HBV seromarker distribution of non-resolved donations, the remaining non-resolved and resolved negative donations.

Seromarkers	Non-resolved donations (%)	Remaining non-resolved donations (%)	Resolved negative donations (%)	P
	N=252 (42 pools)	N=150 (25 pools)	N=85*	
Anti-HBc+	134 (53.2)	79 (52.6)	38 (44.7)	0.15
Anti-HBc+/anti-HBs+	116 (46)	68 (45.3)	37 (43.5)	0.699
Anti-HBc+/ anti-HBs−	18 (7.1)	11 (7.3)	1 (1.2)	0.131
Anti-HBc−	118 (46.8)	71 (47.4)	47 (55.3)	0.723
Anti-HBc−/ anti-HBs+	71 (28.2)	43 (28.7)	29 (34.1)	0.740
Anti-HBc−/ anti-HBs−	47 (18.6)	28 (18.7)	18 (21.2)	0.90
Total(%)	252 (100)	150 (100.0)	85 (100.0)	

*Donations were identified negative from 17 resolved pools. The rates of anti-HBc alone in non-resolved donations (42 pools) and in the remaining non-resolved donations (25 pools) were significantly higher than that in resolved negative donations (P = 0.032 and P = 0.033, one-sided fisher’s exact).

### Genotyping and Mutation Analysis of the S and BCP/PC Regions in the Identified HBV DNA+ Donations

Totally, 17 samples (17/42, 40.5%) in the non-resolved pools were identified HBV DNA+ and classified as OBIs by Ultrio plus and additionally serological and molecular assays. The maximum and median viral loads were 37.2 IU/ml and 5.1 IU/ml, respectively. The phylogenetic analysis identified seven donations with genotype B and one genotype C and one genotype D. The S region amino acid sequences of these nine cases showed that all cases had amino acid substitutions. Regarding the seven genotype B samples, two out of seven (28.6%) samples were observed as wild type (L017 and Q84). Four samples (57.1%) had vaccine escape mutations: G112R (Q8), T126S (L012), M133T (Q27), D144E (Q139), and S174N (Q139). Sample Q27 with anti-HBs harbored a T131N/M133T N-glycosylation mutation, which may interfere with recognition of HBsAg by anti-HBs, therefore contributing to virus escape from the host immune system (15). In addition, 4/7 (57.1%) have mutations that have potential impact on the detection of HBsAg: T126S (L012), M133T (Q27), F134L (Q27), T143L (Q8), L175S (Q139). Various mutations associated with OBIs—Q101R (N022 and Q139), P105R (Q139), T126S (L012), P127H (Q27), M133T (Q27), S174N (Q139), and V177A (Q139)—were also detected in four (57.1%) samples. For the one genotype C sequence, it harbored multiple mutations including Q30K, L53S, T113K, and T118K, and lastly a genotype D sample carrying V96A, L104W, G112E, P127L, Q129P, and S164N mutations.

BCP/PC genes were amplified from six samples by nested PCR. Nucleotide mutations with high frequency were found as follows: 5/6 (83.3%) sequences contain T1719G, which has been previously reported to inhibit HBV replication through Enh II and HBx proteins mutation *in vitro* (16). Also present were 6/6 (100%) A1726C and 5/6 (83.3%) C1730G mutations. In addition, other mutations such as A1752G/T (33.3%), C1773T (33.3%), G1809T (33.3%), A1846T (33.3%), G1799C (16.7%), C1853T (16.7%), G1896A (16.7%), G1899A (16.7%), and G1915A (16.7%) were also observed.

## Discussion

Donor screening is essential to ensure blood safety to prevent transfusion-transmitted infections. To decrease the residual risk for HBV infection, both HBsAg and anti-HBc, together with highly sensitive HBV NAT (ideally ID-NAT) screening, provide the highest level of blood safety for recipients, but this comprehensive screening strategy is only adopted in high income countries due to high cost and strict requirement of NAT (17). In most developing countries, especially China with a high positive rate for anti-HBc, alternative approaches can be used to balance the cost and safety. Currently HBsAg and MP-NAT screening have been used in China to prevent most cases of HBV infections from transfusion. In countries where NAT screening is not implemented, screening HBsAg and anti-HBc could identify donors with OBI at relatively low cost. Then, testing donations with HBsAg negative but anti-HBc positive using a highly sensitive NAT for HBV-DNA could intercept most acute HBV infections within window period (WP) or OBIs. These two different safety procedures applied in sequence could guarantee blood safety at a relatively low cost. As a compromised strategy, most Chinese blood centers adopt both HBsAg and HBV MP-NAT screening to balance the cost and safety by shortening the WP and intercepting most OBIs. The same approach has also been applied in Taiwan (18). In a country with a high positive rate for anti-HBc, the use of anti-HBc positive/HBsAg negative/HBV-DNA negative donations for anti-HBc (>100 UI) and/or HBsAg positive individuals should also be considered if blood shortage exists. Although blood screening strategy has been improved, problems still exist, especially in many countries with high prevalence of blood-borne pathogen infections. While adopting NAT assays greatly shorten the window period that allows detection of many serologically undetectable infections possible, donations with MP-reactive but ID-non-reactive donations (MP+/ID−, defined as non-resolved donations) have been observed during daily screening process. These non-resolved donations may pose a significant risk for transfusion-transmitted infections in recipients, especially in China where HBV infection is endemic and anti-HBc testing cannot be implemented in routine blood screening. A Chinese multicenter study performed on 826,044 serologic negative donations in MPs of six identified that a total of 1,267 pools were reactive, of which 839 donations were reactive by ID-NAT, leaving 428(33.8%) non-resolved MPs (19). These NAT initially reactive (IR)/non-resolved MPs might contain plasma from anti-HBc+ OBI donors with extremely low and intermittently detectable HBV DNA load but still potentially infectious (17, 19), raising urgent need for a practical algorithm to sort out which blood units can be safely transfused.

Previously, we reported nearly half of the initial Ultrio plus NAT reactive, but further discriminatory test negative donations were identified as OBIs in ID-NAT screening setting (12), strongly suggesting a large proportion of samples had viral load below the 95% LOD of the Ultrio Plus ID-NAT assay (LOD: 6.8 IU/donation) and also the MPX2.0 MP6 NAT assay (LOD: 13.8 IU/donation). Donations with such low viral loads had a high probability of being missed by the subsequent HBV ID-NAT, and this probability was determined by Poisson distribution (20). The 5% LOD of the MPX assay is higher when testing in MP6 than in ID format (the 95% LOD of MPX2.0 test: 2.3 IU/ml [ID format] and 13.8 IU/ml [MP format]) (10), but a dilution factor of six is relatively small on the whole NAT detection endpoint probability curve that spans a concentration range of a factor of 100 between the 95 and the 5% LOD (19). Thus, it is likely to have a reactive result in a pool of six samples but not in any of the individual samples. Another possibility of MP-NAT reactive but ID-NAT non-reactive (non-resolved) donations may be due to contamination, although this possibility is very low. To avoid contamination, we established a NAT laboratory with international standard, and all the screening procedures are fully automatic, even for screw capping. Furthermore, we adopted strict standard biosecurity and institutional safety procedures during the screening process.

In this study we screened 17,226 pools (103,356 donations) by Roche MPX2.0 MP6-NAT, and we identified 98 (0.57%, 95% CI 0.46–0.69%) MPs were initial reactive, among which 56/98 (57.1%) were resolved HBV DNA+. HBV DNA+ rate is 0.054% in this study cohort and is lower than screened by ID-NAT format reported in our previous study (12), likely because of dilution factor in MP-NAT and low-viral-load donations. Forty-two of 17,226 (0.24%, 95% CI 0.18–0.33%) MPs were initially reactive, but all six donations from each MP were non-reactive (designated as non-resolved) when tested individually. This non-resolved percentage is in concordance with the study in Australia (21), but it is lower than the result from a national survey (19), probably due to the fact that the implementation of NAT in routine blood screening in Shenzhen Blood Center was 10 years earlier than other Chinese blood centers. After 42 non-resolved pools were further tested by Ultrio Plus ID-NAT, seven donations from 42 MPs (252 donations) were identified HBV DNA+ with anti-HBc+, and they became resolved. Since the probability of detection by NAT in low-viral-load samples follows a Poisson distribution, we tested a total of 210 (0.20%) donations in the remaining 35 non-resolved pools by 2.5 ml large-volume extraction followed by nested PCR amplification and qPCR, and 10 donations from 35 MPs (210 donations) were further identified HBV DNA+ with anti-HBc, indicating they were OBIs. It is undoubted that some low-viral-load donations were detected in MP6 format but missed by ID-NATs. To sum up, 17 donations from 17/42 (40.4%) non-resolved pools (252 donations) containing HBV DNA+ were detected by additional alternative NATs, leaving 25/98 (25.5%) remaining non-resolved pools including 150 donations. These donations were released and transfused to recipients, posing potential threat to blood safety.

Previous studies estimated OBI transmission rate for all components varied between 3 and 48% (8, 22, 23), which might be underestimated. A recent mathematical model estimated that 3.3 and 14% of OBI donations undetected by NAT with LOD of 3.4 IU/ml might cause recipient infection by a blood component containing 20 ml and 200 ml of plasma, respectively (24). Infectious donations not detected by MP-NAT but reactive with ID-NAT have been reported (25, 26). According to clinical evidence and sequence identity, HBV transfusion transmission in 9/31 recipients (29%) of blood components from donations undetected by the currently most sensitive NAT and HBsAg showed that even low levels of DNA in donors can be infectious, and the revised lowest infectious dose was down to 0.14 IU/ml (7). Furthermore, two cases of transfusion-transmitted HBV infection were identified by donor-recipient sequence identity following transfusion of 14 OBI donations missed by MP6 HBV DNA screening (8). In ID-NAT screening, NRR donations are not released for transfusion in most countries. However, for MP6 NAT, it is not an option to discard the units implicated in non-resolved pools. In this study, we used large-volume viral nucleic acid extraction, together with the highly sensitive nested PCR, to detect viral fragments, and we identified that 40% of non-resolved pools contained HBV DNA with anti-HBc. These donations could transmit HBV especially in immune compromised recipients. Our results indicated that even after MP-NAT screening in China, donations with OBI still pose a potential residual threat for blood safety. Although transfusion-transmission evidence is needed to substantiate this risk and anti-HBc and anti-HBs prevalence in recipients should also be considered, our data still suggest that a proportion of non-resolved pools still contain extremely low levels of HBV that may be infectious, and more comprehensive resolving strategy should be considered for MP-NAT.

The presence of anti-HBs in addition to anti-HBc indicates a resolved infection with persistent HBV DNA (27). In some countries such as Germany and Austria, blood units with anti-HBs levels greater than 100 IU/L are considered to be safe (28), and in Japan, anti-HBc-positive blood containing 200 IU/L or more of anti-HBs appears safe as a transfusion component (29). However, transmission of HBV from occult hepatitis B subjects occurred in the presence of concurrent neutralizing anti-HBs in the same specimen (30, 31). Data from organ transplantation also clearly proved that HBV DNA in the presence of anti-HBs could be infectious in immunosuppressed patients (32). HBV DNA detected in some anti-HBs-positive samples in this study suggests that the absence of HBsAg and the presence of anti-HBs do not necessarily guarantee full safety. In the present study, 74.2% donors were anti-HBs+ including 46% with anti-HBc and 28.2% anti-HBs only, suggesting only a small part of donors (28.2%) had a certain protection; most were induced by immune response when non-vaccinated donor exposed to HBV or an anamnestic response to HBV when vaccinated donor exposed. Eight of 17 (47.1%) OBIs detected in non-resolved donations carry anti-HBs, suggesting that OBIs occur largely in individuals who have recovered from the infection but are unable to develop a totally effective immune control (33, 34). Anti-HBc alone has been observed either in a stage of late HBV immunity after the decline of anti-HBs to undetectable levels or in the resolving phase of acute infection. Consistent with OBI, donated samples carrying anti-HBc alone are more infectious than those with low levels of anti-HBs (35). Even certain PCR negative “anti-HBc alone” individuals have been suspected to be potentially infectious (36). According to a recent study, blood donors negative for both HBsAg and HBV DNA but reactive for anti-HBc might be HBV carriers with viral loads below the detection limit (37). In our present study, 8/17 non-resolved donations were identified as OBIs with anti-HBc alone, and these OBIs may pose significant threat to blood safety.

It is well-known that anti-HBc is detectable during asymptomatic infections as well as throughout life after recovery from HBV infection with or without the presence of anti-HBs (38); therefore, anti-HBc is considered a key seromarker for OBI. Anti-HBc screening assays have the potential to exclude the majority of OBIs undetectable by NAT (27, 39, 40), leaving only rare cases with escape mutants associated with the presence of anti-HBs alone (41). Many studies supported the use of serological markers such as anti-HBc to compensate less-sensitive NAT assays (42, 43). However, only a relatively small portion of OBIs can be identified by MP-NAT, which emphasizes the importance of anti-HBc testing and ID-NAT screening. In line with this, an American comprehensive study from 22.4 million blood donors screened by HBsAg, anti-HBc, and NAT revealed that only 43/404 (10.6%) OBIs could be detected by MP-NAT, while most of OBIs (361/404, 89.4%) could only be identified by ID-NAT (44). These results indicated that the potential relative risk of OBI among MP- positive donations may be small compared to that in MP-negative donations. Nevertheless, in countries such as in China, where HBV endemic infections are high, anti-HBc screening may cause blood shortage. Considering the cost, ID-NAT is not a mandatory requirement for donor screening in China; therefore, an alternative approach is to screen MP-positive donations with anti-HBc to identify those donations with potential OBIs to balance the cost and safety. Ideally, blood centers should adopt ID-NAT screening especially in regions with high HBV endemicity, but in reality, different strategies have been used. We made a rough analysis of cost-effectiveness by comparing three strategies: (1) HBsAg+ anti-HBc, (2) HBsAg+ MP6 NAT, (3) HBsAg+ MP6+ anti-HBc in MP+/ID− (**Table 4**). Undoubtedly, HBsAg and anti-HBc screening is the most highly cost-effective, but it would overkill about 40% anti-HBc+ donations, resulting in blood shortage. Adding anti-HBc screening for MP+/ID− non-resolved donations would cost 5,004 RMB more but at least benefit from preventing the occurrence of six transfusion-transmitted HBV cases (**Table 4**) with only deferring 0.11% anti-HBc+ donations to transfusion, but obtaining 67.6 times benefit (46).

**Table 4:** Cost-effectiveness analysis of 103,955 donors* in Chinese Shenzhen Blood Center.

	Screening strategy		
	HBsAg+anti-HBc	HBsAg+ MP6 NAT	HBsAg+ MP6+ anti-HBc in MP+/ID−
Cost (RMB)	1,247,460	1,435,748	1,436,252
CHB^a^	249	249	249
OBI^b^	194	56	73
WP acute infection	0	1.8	1.8
Predicted transfusion transmitted HBV cases^c^	272.8	224.8	231
Benefit (RMB)	114,576,000	94,416,000	97,020,000
Benefit/cost	91.8	65.8	67.6

*After screening 103,955 donors by ELISA, 103,356 seronegative blood samples (17,226 MP6 pools) were enrolled in this study.

HBsAg DiaSorin (Italy) ELISA: 10 RMB/test; WanTai anti-HBc ELISA: 2 RMB/test. 1$=6.9RMB.

MP6: Roche MPX2.0 NAT for HBV DNA/HCV RNA/HIV RNA/ID format: 68 RMB/test, screening for HBV using MP6: 23 RMB/pool (17,226 pools).

CHB: the rate of HBsAg ELISA+ was 0.3% in Shenzhen blood donors’ population, of which 80% were NAT+, and confirmed HBsAg+/DNA+ as CHB.

OBI: according to previous study (12), we predicted the OBI with anti-HBc were (113+162×72.6%) × 103955/123280 = 194. The percentage of OBI with anti-HBc in NAT+ was 72.6% (45).

Transmissions rate of OBI by blood transfusion = 2/11 (18.2%) (11 donor-recipient pairs cause two HBV infections (8). Transmissions rate of WP acute HBV infection by blood transfusion = 63% (23). Transmissions rate of CHB by blood transfusion = 40.6% (median of OBI and WP, no reference data).

C = (a×0.406+b×0.182+c×0.63) × 2 (a donation produces two units washed red blood cells and 200 ml frozen plasma, at least transfused two recipients).

Benefit (RMB)= Predicted transfusion transmitted HBV cases×420,000 (46), preventing one case of HBV infection can recover the medical cost of 420,000 RMB.

It has been reported that mutations in S gene or promoter and enhancer sequences of HBV genome could result in false negative in HBsAg detection and NAT assays (47, 48). Although mutations from the consensus (wild type) may also be present in a minority of non-OBI sequences, according to our sequence analysis, some OBIs indeed carry mutations in S gene or in BCP/PC, which most likely affect HBsAg detection (T126S, M133T, F134L, T143L, L175S) or inhibit HBV DNA replication (T1719G). The mutation impact remains speculative without functional analysis, and we are in the process of setting up these assays to further confirm the biological significance of some of these mutations, especially in evasion of immune surveillance and detection.

Although more specific and sensitive NATs are urgently needed for donor screening in MP format, it is a long way to guarantee blood safety. As the majority of individuals with OBI have very low viral loads together with various mutations, the application of anti-HBc testing to evaluate non-resolved donors provides a better way to enhance blood safety in China. Additionally, some sensitive molecular methods like nested PCR and real-time PCR assay with high extraction volume or nucleic acid hybridization for HBV DNA should be applied to identify OBIs in non-resolved blood donors.

## Data Availability Statement

The original contributions presented in the study are included in the article/supplementary material, further inquiries can be directed to the corresponding author.

## Ethics Statement

This study was reviewed and approved by the ethics committee of the Shenzhen Blood Center. The written and informed consent form was obtained from each donor before donation.

## Author Contributions

XY designed the experiments and wrote and reviewed the manuscript. LC reviewed, revised, and edited the manuscript. YZ, RL, TL, XZ, WX, JZ, MX participated in the study design, performed the experiments, and collected and analyzed the data. All authors contributed to the article and approved the submitted version.

## Funding

This work was supported by the Nature and Science Fund of Shenzhen and Guangdong (JCYJ20190806112201646 and 2021A515010979) and Shenzhen Key Medical Discipline Construction Fund (SZXK070) to XY and CAMS Initiative for Innovative Medicine (CAMS-2016-I2M-3-025 and CAMS-2017-I2M-B&R-15), National Key Research and Development Program (2018YFE0107500), Science and Technology Partnership Program, Ministry of Science and Technology of China (KY201904011) to LC.

## Conflict of Interest

The authors declare that the research was conducted in the absence of any commercial or financial relationships that could be construed as a potential conflict of interest.

## Publisher’s Note

All claims expressed in this article are solely those of the authors and do not necessarily represent those of their affiliated organizations, or those of the publisher, the editors and the reviewers. Any product that may be evaluated in this article, or claim that may be made by its manufacturer, is not guaranteed or endorsed by the publisher.

## Abbreviations

HBV, Hepatitis B virus; HCV, hepatitis C virus; HIV, human immunodeficiency virus; HBsAg, hepatitis B surface antigen; anti-HBc, hepatitis B core antigen; anti-HBs, hepatitis B surface antibody; HCC, hepatocellular carcinoma; NAT, nucleic acid testing; ELISA(s), enzyme-linked immuno-absorbent assay(s); ECLI, electrochemiluminescence immunoassay; ID, individual donation; LOD, limit of detection; MP(s), mini pool(s); ID, individual donation; NRR, non-repeat reactive; MP6, mini pools of six donations; OBI, occult hepatitis B virus infection; BCP/PC, basic core and pre-core promoter regions.
